# Characteristics of Wind Velocity and Temperature Change Near an Escarpment-Shaped Road Embankment

**DOI:** 10.1155/2014/695629

**Published:** 2014-07-20

**Authors:** Young-Moon Kim, Ki-Pyo You, Jang-Youl You

**Affiliations:** ^1^The Department of Architectural Engineering, Chonbuk National University, Jeonju 561-756, Republic of Korea; ^2^Long-Span Steel Frame System Research Center, Chonbuk National University, Jeonju 561-756, Republic of Korea; ^3^KOCED Wind Tunnel Center, Chonbuk National University, Jeonju 561-756, Republic of Korea

## Abstract

Artificial structures such as embankments built during the construction of highways influence the surrounding airflow. Various types of damage can occur due to changes in the wind velocity and temperature around highway embankments. However, no study has accurately measured micrometeorological changes (wind velocity and temperature) due to embankments. This study conducted a wind tunnel test and field measurement to identify changes in wind velocity and temperature before and after the construction of embankments around roads. Changes in wind velocity around an embankment after its construction were found to be influenced by the surrounding wind velocity, wind angle, and the level difference and distance from the embankment. When the level difference from the embankment was large and the distance was up to 3*H*, the degree of wind velocity declines was found to be large. In changes in reference wind velocities around the embankment, wind velocity increases were not proportional to the rate at which wind velocities declined. The construction of the embankment influenced surrounding temperatures. The degree of temperature change was large in locations with large level differences from the embankment at daybreak and during evening hours when wind velocity changes were small.

## 1. Introduction

When a highway is constructed in a mountainous area, highway embankments and tunnels may be introduced. Highways constructed in mountainous areas using such methods may damage agricultural products by disturbing the natural sunlight and ventilation of an area. When an artificial structure such as an embankment is constructed in a “fruit” farming area, the disturbance of natural airflow can cause the temperature of the area to change, which can result in damage such as the withering away of fruit trees, reduced crop yields, and delays in blooming, all of which reduce the quality of the crops. Although wind corridors can be installed around embankments to prevent the damage of cold weather caused by cutting off the airflow, they have not been particularly effective. Most of the cold-weather damage to fruit trees in areas with highway embankments occurs in the spring when the wind is weak. This is because most cold-weather damage is caused by poor airflow. Particularly, when slopes are being constructed on valley-shaped open land, the free flow of air is blocked by highway embankments, and the temperature in the area becomes lower than that in other neighboring areas, enhancing the cold-weather damage. Airflow changes in sloped areas are more complicated and more diversified due to topographic effects.

There are many phenomena unique to different topographies, such as gusting and wind velocity increases and decreases caused by covering effects. Wind velocity is increased on a slope, and it may be increased by certain other topographic effects of the land. Many studies have examined the increase in wind velocities in mountainous, valley, and sloped areas. Both Jackson and Hunt [1] and Mason and Sykes [2] studied the effects of wind velocity increases in lower mountainous areas without separation phenomena. Bowen [3] studied wind velocity in simple two-dimensional mountainous areas. Tayor and Lee [4] proposed an algorithm to forecast wind velocity increases at the peak of a mountainous area. Most studies have focused on the distribution of wind velocities under various warm current conditions in mountainous areas (Newley [5], Neff and King [6], Finardi et al. [7], Booij et al. [8], and Vosper et al. [9]). Miller and Davenport [10] and Li et al. [11] performed comparative analyses on wind velocity increases in complex mountainous areas by considering surface roughness suggested in major loading criteria and surrounding geographical features. In addition, they emphasized surface roughness and surrounding air current conditions when predicting wind velocity increases. Weng et al. [12] proposed guidelines on air currents in complex geography by considering geographical features and surface roughness. Svoboda and Čermák [13] measured the wind velocities and their distribution in the ridges of the Erzgebirge Mountains using Doppler Sodar observations. Chock and Cochran [14] performed a wind tunnel test to investigate the rate of the wind increase phenomenon on an island with a varied topography and proposed an experimental model regarding the peak and wind velocity increase that could be applied to the design of field structures.

However, highway embankments influence the lower currents at the bottom of a slope. There have not been sufficient studies carried out on airflow near an artificial structure such as a highway embankment. As fruits grown in the bare ground at the bottom of a slope are sensitive to temperature and wind velocity, wind velocity and temperature should be evaluated before constructing roads on the slope. In this study, wind velocity and temperature changes before and after the construction of highway embankments on valley-shaped open land were investigated. For changes in wind velocity, a wind tunnel test was performed using models. In the wind tunnel test, a model was used to identify the wind velocity change before and after the construction of highway embankments. The correlation between the wind velocity and temperature near highway embankments was identified when the field experiment in the highway embankment's neighboring areas was conducted.

## 2. Location and Study Method

**Figure 1:**
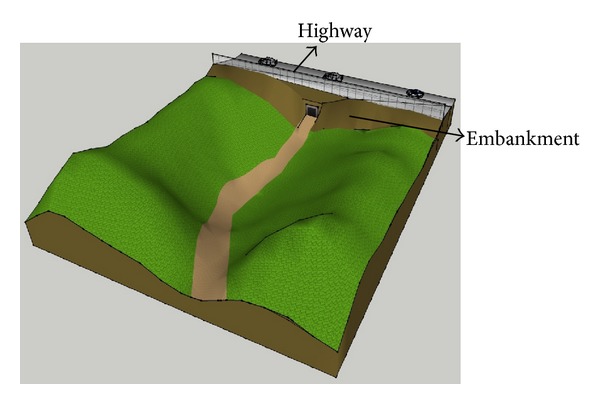
Topographic map of area surrounding embankment.

In the test site, fruit farms were distributed around an area comprised of a 1.5 km embankment in a highway construction section. The highway embankments, located at 36° 3.4′N and 140° 7.5′ (E), and their surrounding areas are shown in Figure 1. Before the construction of the embankments, air could naturally flow down to the bottom of the mountain. However, it seems that the construction of the embankments affected the airflow. To evaluate wind velocity and temperature change in the areas surrounding the highway embankments, two types of tests were executed. First, by making a miniature land model, a wind tunnel test was performed to identify wind velocity changes in survey points before and after the construction. Second, a field experiment was performed to identify the correlation between temperature and wind velocity changes in the fruit farming area after implementation of the embankment.

## 3. Wind Tunnel Test

### 3.1. Experimental Model

**Figure 2:**
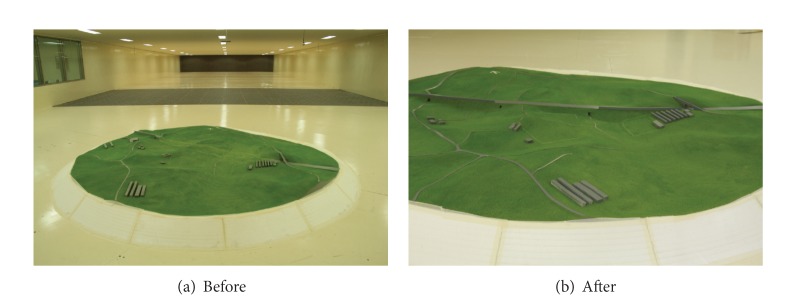
Land model installed inside wind tunnel.

**Figure 3:**
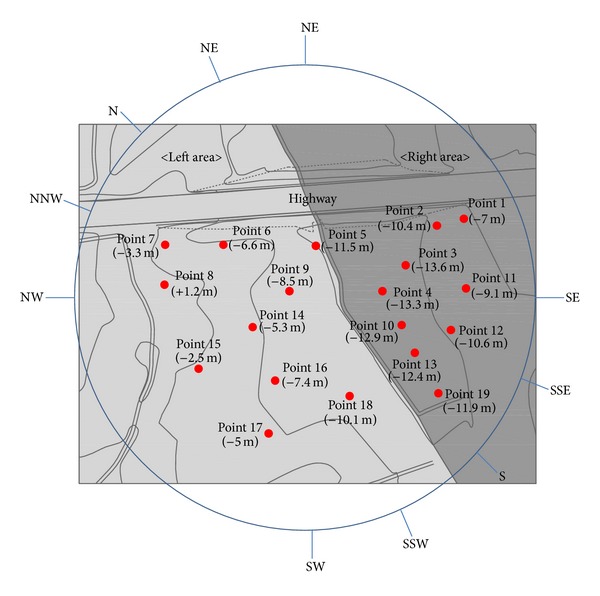
Wind angles and measurement points.

To identify the flow of air near the highway embankments, a wind velocity test was performed upon a 1/150 scaled land model. The land model for the wind tunnel was made of Styrofoam, and an aluminum bar was installed so that anemometers could be installed to measure wind velocity. The turbulent boundary layer wind tunnel device was a vertical circulation closed-circuit type, and the sectional size of the tunnel was 12 m in width, 2.5 m in height, and 40 m in length.  Figure 2 shows the experimental land model installed inside the wind tunnel. To identify wind velocity changes in highway embankments, multichannel anemometers (System 6242 Model 1560) were used. The experiment was performed to identify changes in wind velocity according to the level difference in the surrounding topography before and after the construction of highway embankments with a certain initial wind velocity. To identify changes in wind velocity below the embankment, a total of 19 points were selected, as there was a difference in the levels from the southern side and northern side of the embankment. The anemometer was installed only in the southern direction. As the southern area was larger than the northern area, it was used as an orchard. Wind velocity tests were conducted in five sites just below the embankment and in 14 sites at random distances from the embankment. The tested wind angles were limited to those of the winds blown from the northern and southern directions of the embankment. The wind velocity tests were performed on 10 wind angles including each set of four wind angles with a 22.5° gap between the NW-NE wind angle and the SW-SE wind angle.  Figure 3 presents the wind angles in the wind tunnel test. The anemometer that measured reference wind velocities was installed above the road with the embankment. The heights of 19 measurement sites and the reference anemometer were fixed at 10 mm (full-scale height was 1.5 m).

### 3.2. Wind Velocity Measurement Results

Three reference wind velocities were used in measuring wind velocities: 3 m/s, 5 m/s, and 7 m/s. The reference wind velocities were based on the wind velocities measured by the anemometer on the embankment road. This test examined wind velocity changes by measurement site according to changes in the reference wind velocities in the surrounding area before and after the construction of the embankment.

Figure 3 shows the level difference based on the measurement sites to measure wind velocities around the embankment and the height of the embankment road. The adjacent area below the embankment had an average level difference of −8.5 m. Based on the central point of the embankment, the left area had the largest level difference of −11 m, and the right area had a level difference of −5.9 m.

**Figure 4:**
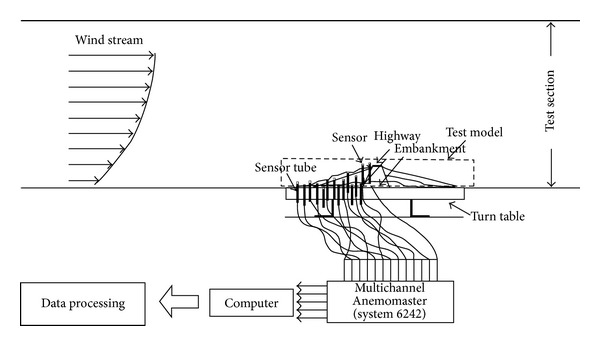
Outline of measurement of wind velocity.

**Figure 5:**
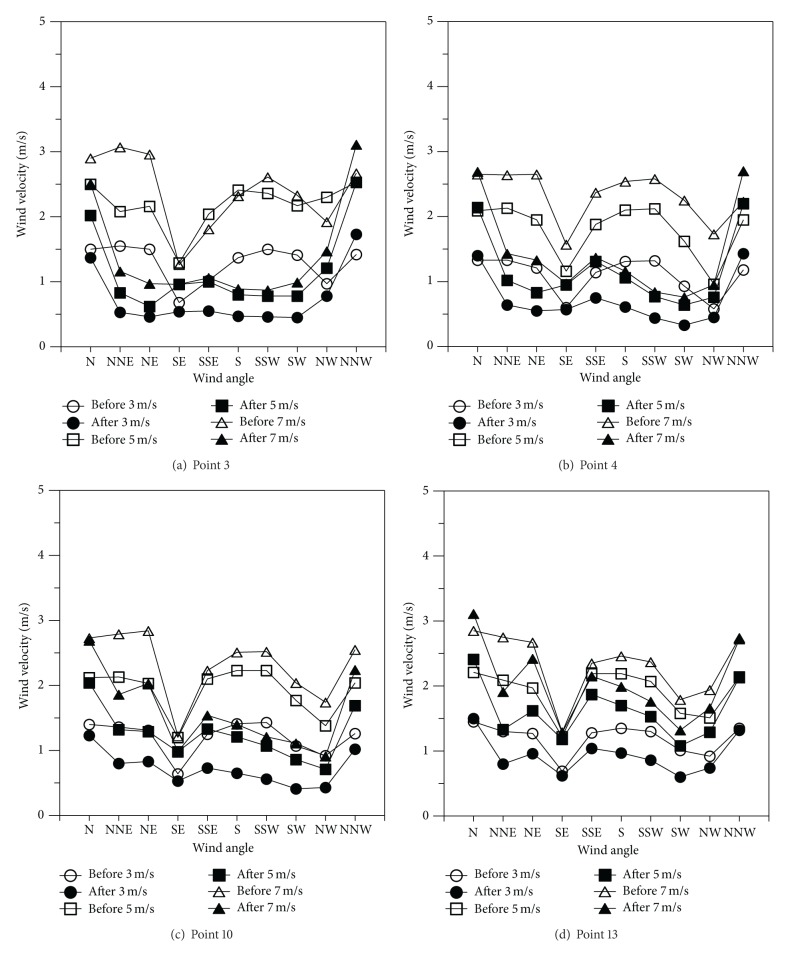
Distribution of wind velocities by measurement site according to wind angle changes in area to right of embankment.

Figure 4 shows the outline of the wind velocity measurements by reference wind velocity and measurement site.  Figure 5 shows the distribution of wind velocities by measurement site according to wind angle changes in the area to the right of the embankment. Wind velocity changes by measurement site were found to vary compared to the reference wind velocities according to wind angle changes. However, the wind velocity of the southeastern position as the valley wind on the land was at most 60% smaller than the wind velocities measured from other wind directions. After the construction of the embankment road, large declines in wind velocity were shown compared to the reference wind velocities in all measured wind directions except some northern directions. The wind directions (N and NNW) with small wind velocity changes before and after the construction of the embankment were found in the sites with lower embankment heights than the other sites. This study examined wind velocity changes according to increases in the reference wind velocities before and after the construction of the embankment. The wind angles from some northern directions (N, NNW, and NW) before and after the construction of the embankment showed that the rates of decline of wind velocities after construction were small at less than 20% regardless of the measurement location or wind velocity. A smaller distance between the measurement site and the embankment and an increase in the reference wind velocity resulted in a corresponding larger degree of wind velocity decline. This study examined wind velocity changes compared to the reference wind velocities according to the level difference between the embankment's height and the measurement site. In the case of Measurement Site 3, it was −13.6 m under the embankment road. After its construction, wind velocity changes were 1 or below in all wind velocities. It was confirmed that the rate of decline of wind velocities was being influenced by the level difference from the embankment.

**Figure 6:**
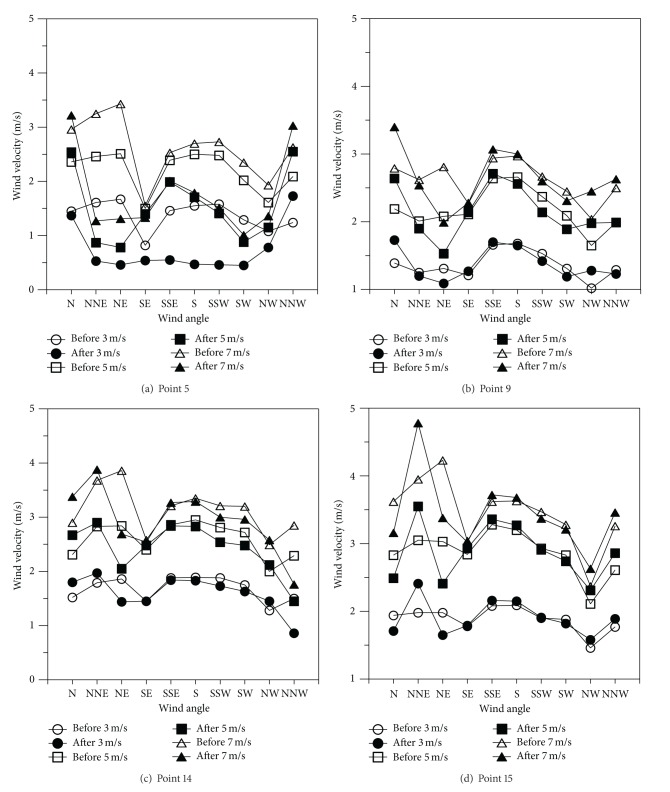
Distribution of wind velocities by measurement site according to wind angle changes in area to left of embankment.

Figure 6 shows wind velocity changes by measurement site according to wind angle changes in the area to the left of the embankment. The left area contained many areas that were over 50% higher in terms of the average level difference. The left area was also being influenced by the measurement sites and wind angles in the degree of wind velocity changes compared to the reference wind velocities before and after the construction of the embankment. Measurement Site 5 located just below the embankment had a level difference of −11.5 m from the embankment road and showed a large degree of wind velocity decline with over 70% after the construction of the embankment at a reference wind velocity of 3 m/s. However, Measurement Sites 9, 14, and 15 exhibited little wind velocity changes compared to the reference wind velocities regarding the wind angles of the southern direction before and after the construction of the embankment. This is probably because these sites had larger level differences than the equivalent right sites. It was confirmed that the wind velocity changes around the embankment were largely influenced by the distance and level difference from the embankment.

**Figure 7:**
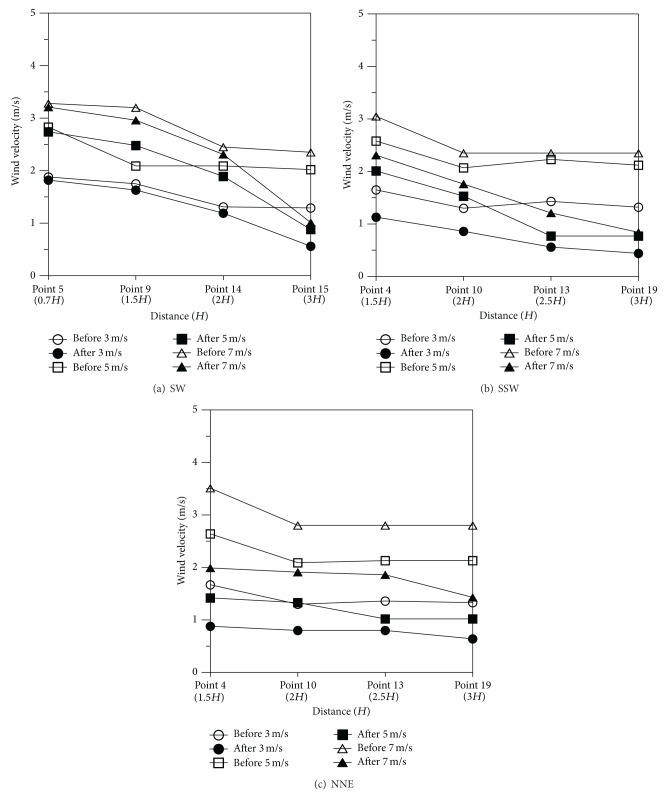
Wind velocities per minute by embankment's distance according to wind angle changes.

**Figure 8:**
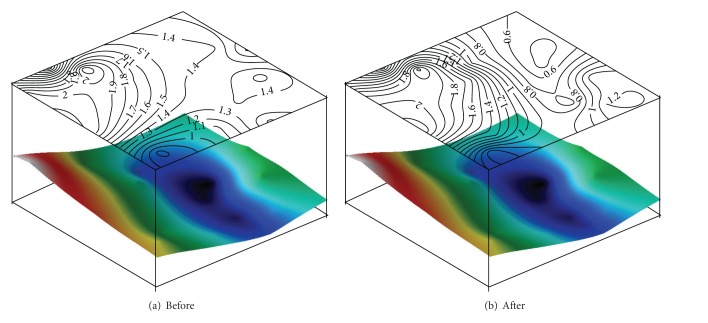
Overall wind velocity distribution chart within location before and after construction of embankment (wind angle = SSW).

Figure 7 shows wind velocity changes by distance from the embankment according to wind angle changes. Before the construction of the embankment, wind velocity changes by distance were shown to be consistent without large influences from wind angles. However, after the construction of the embankment, wind velocity changes compared to the reference wind velocities according to the distance from the embankment were confirmed to be influenced by wind angles. In wind velocity changes by the measurement distance of the wind angles SSW and SW, the site that was 3*H* (*H* = the embankment's height) away from the embankment showed a decline of wind velocity ratios of up to over 60% compared to the site 1.5*H* away from the embankment regardless of wind velocity changes. However, in the wind angle NNW blown from the embankment's north, there were no wind velocity changes by distance. Wind velocity changes by distance from the embankment were influenced by wind angles.  Figure 8 shows the wind velocity distribution of the area surrounding the embankment when the wind was blowing from SSW at 3 m/s.

Figure 8 below the wind velocity distribution shows the field topography by color. Areas with lower altitudes are shown in black, and higher altitudes are shown in red. Before inserting the model slope, the wind velocity was distributed according to topography. Therefore, the left area, which had higher topography, always had at least 2 m/s of wind velocity. In the lower level, there was always at least 1.35 m/s of wind velocity. However, when highway embankments were constructed, the right area with its lower topography had a more than 55% wind velocity reduction, which reduced the wind velocity to less than 1 m/s. There was no significant reduction in wind velocity in the left area with a smaller level difference.

## 4. Field Experiment

**Figure 9:**
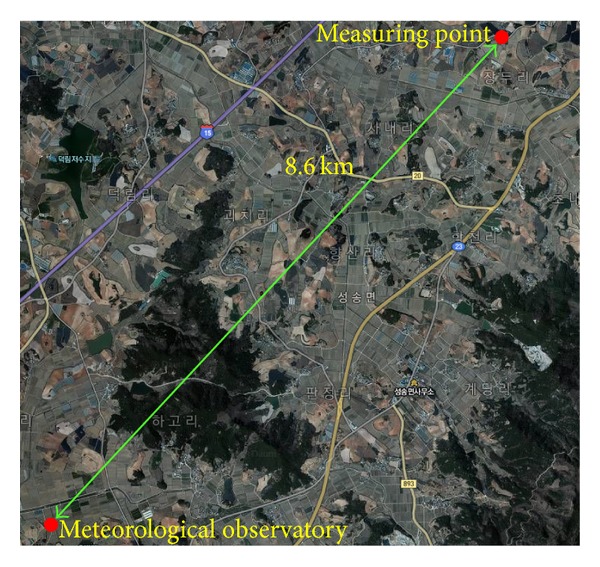
Distance between meteorological observatory and field-measured points.

**Figure 10:**
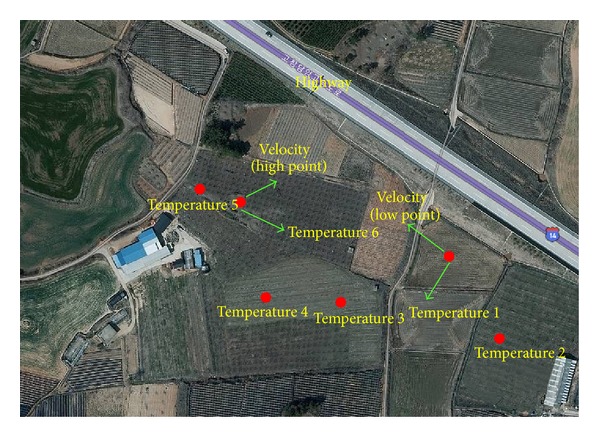
Field-measured points.

To identify the correlation between the surface wind velocity and the temperature change in the area of highway embankments, a field experiment was performed.  Figure 9 shows the distance between the meteorological observatory and the field experiment site (8.6 km in a straight line from the measured points). The field experiment was conducted based on an average temperature of 5.6°, maximum temperature of 21.4°, minimum temperature of −4.1°, and average wind velocity of 3.4 m/s in March (as observed in the nearest meteorological observatory). In the field experiment, wind velocity and temperature distribution were identified focusing on the lowest point (−11.5 m) and the highest point (1.2 m) of the embankment.  Figure 10 shows the location of the field experiment site. To identify changes in wind velocity and temperature according to the height of the embankment, anemometers were installed at the highest and lowest points.

**Figure 11:**
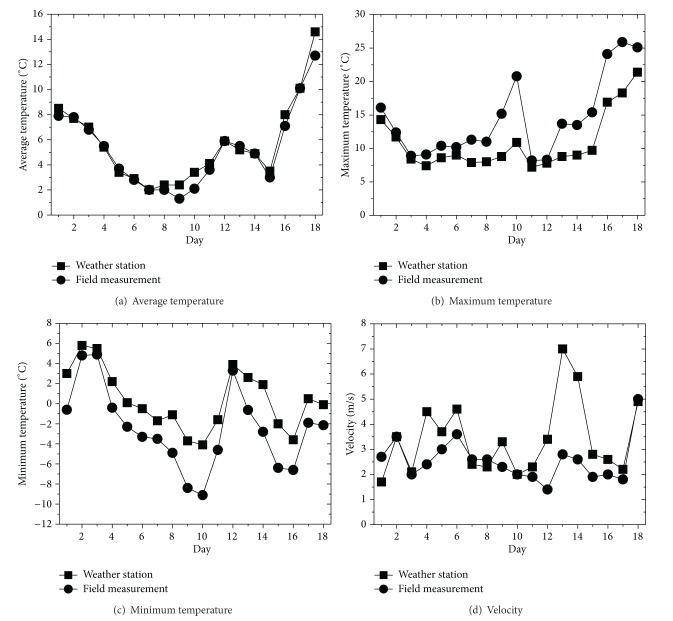
Wind velocity change according to wind angle change by measured points before and after construction of embankment.

**Figure 12:**
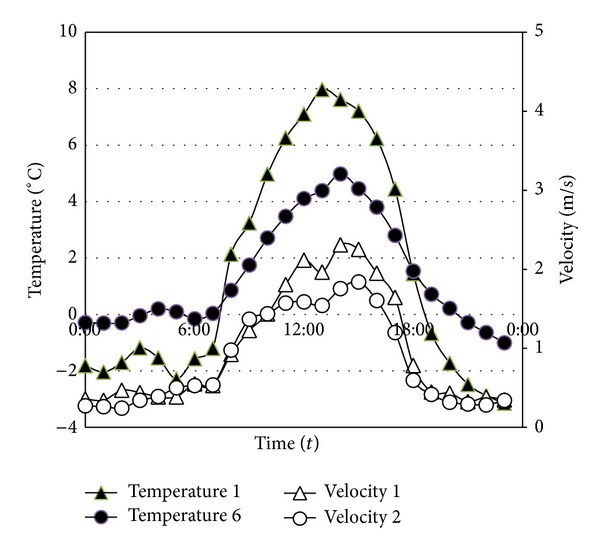
Temperature and wind velocity distribution at measured points by time zone during measurement period.

Five points between the two anemometers were selected as temperature measure points. The temperature change was recorded for 18 days, and the average temperature data measured every 5 minutes was automatically saved. The measurement range of the temperature sensor (HOBO Pro v2 Tem/RH Data Logger) was −40–70°C, and the measurement range of the wind velocity sensor was 0.5–50 m/s.  Figure 11 compares the temperatures (average, maximum, and minimum) and wind velocities between the data recorded in the meteorological observatory and that measured in the field experiment during the 18-day experimental period. The weather station was 8.6 Km away from the field measurement location in straight-line distance, but their average temperatures were consistent. However, the number of days when a minimum temperature of below 0°C was observed was 9 days according to the meteorological observatory but 15 days in the field experiment, which means that the field-measured points had six more days that showed a minimum temperature of below 0°C. When the average temperature in the meteorological observatory was −4.1°C, it was −9.1°C at the field experiment site. For the average wind velocity distribution, a wind velocity of 1.1 m/s–2 m/s was shown for eight days in the field, while it was shown for only two days at the meteorological observatory. A wind velocity higher than 3 m/s was shown for three days in the field experiment and nine days at the meteorological observatory. The wind velocity was lower in the field-measured points than at the meteorological observatory. When comparing meteorological data between the meteorological observatory and the field experiment site during the experimental period (18 days), it was found that higher temperatures and lower wind velocities were observed more often at the field experiment site, although the highest recorded temperatures were almost identical.  Figure 12 shows the average wind velocity and temperature at points (1.2 m and −11.5 m from the embankment) plotted against time. It was found that the temperature dropped below 0°C as the wind velocity rapidly decreased before 6 am and after 18 pm.

The lowest point in the site (Temperature Measure Point 1) showed a 2°C lower temperature than the other point of the same height on the embankment (Temperature Measure Point 6). Temperature and wind velocity increased from 8 am and reached a peak at 14 pm. Afterwards, both the temperature and wind velocity decreased. However, the temperatures and wind velocities at points lower than the height of the embankment were up to 40% lower than those at points higher than the embankment. From these results, it was confirmed that both temperature and wind velocity were affected by filling in at the field experiment site. In general, temperature distributions by height do not yield large temperature deviations by height on cloudy days due to small amounts of radiation. However, they show large temperature deviations by height on clear and windless days. While the temperatures of low-level sites installed with the embankment were measured at lower levels than those of high-level sites at dawn at below-zero temperatures and in the evening, they were measured at higher levels at noon when the temperature rose. In other words, a temperature-reversal phenomenon was observed.

**Figure 13:**
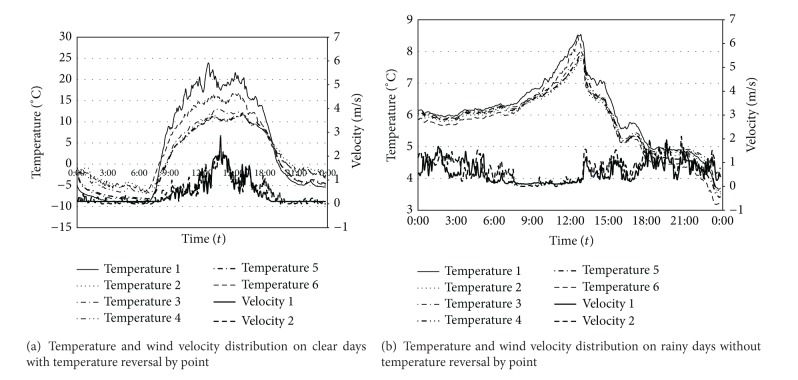
Temperature and wind velocity distribution on clear days and rainy days.

This temperature-reversal phenomenon is shown in Figure 13, which shows a graph of time averages during the measurement period. In the measured data, the temperature in lower areas was 2.0°C lower than in higher areas at night, but it was also 3.5°C higher in the daytime. Figure 13 shows 24 hours' worth of data measured at survey points on rainy days and the days prior to rainy days. In the daytime before rainy days, there was a clear temperature-reversal phenomenon in lower areas. The temperature was below zero in the dawns and evenings and above zero in the afternoons. However, during rainy days, all survey sites showed tiny temperature differences between the day and night of less than 1°C.

## 5. Relationship between Wind Velocity and Temperature Change

**Figure 14:**
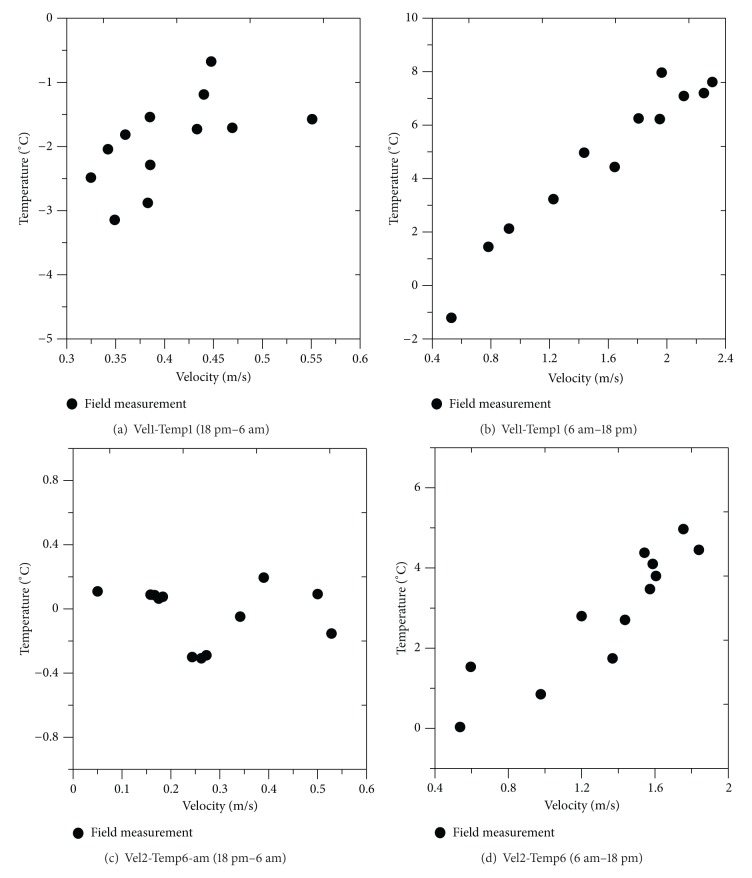
Distribution between wind velocity and temperature by time zone.

The distribution chart of wind velocities and temperatures following the construction of the embankment was examined.  Figure 14 shows the distribution chart of hourly wind velocities and temperatures by experiment site. Based on geographical characteristics, 18 days' worth of data from a high-level site (+1.2 m based on the embankment site) and a low-level site (−13.6 m based on the embankment site) were used. To understand the characteristics of wind velocities and temperature changes, an hourly analysis (18 pm–6 am and 6 am–18 pm) was performed. Wind velocity changes at dawn and during evening hours were very low at below 0.3–0.5 m/s. The low-level site (temperature 1) below the embankment showed temperature changes in the range of 0 to −4°C, while the high-level site showed temperature changes ranging from 0.4 to −0.4°C. The low-level site revealed a larger range of temperature changes than the high-level site. During the hours when the measured wind velocity was very low at 0.5 m/s, the low-level site recorded below-zero temperatures in all temperature ranges. The low-level site's minimal temperature of −4°C showed a temperature difference over ten times that of the high-level site within the same range of wind velocities. During the morning and afternoon hours when the wind velocity was measured at 2.4 m/s or lower, the difference between the maximum and minimum temperatures in the low-level site was 10°C. However, the difference in the high-level site was 5°C. Regarding the characteristics of hourly temperatures, it was confirmed that the embankment reduced the wind velocity and lowered the temperature to the below-zero range. It was also determined that stagnant regions without wind velocity changes due to the embankment influenced the temperature.

## 6. Conclusion

The results of this study regarding wind velocity and temperature changes caused by the embankment around a highway constructed on a sloped topography are as follows.

Wind velocity changes around the embankment were influenced by surrounding wind velocities, wind angles, the level differences of surrounding areas according to the embankment's height, and the distance of areas from the embankment. Wind velocity changes were evaluated in various terms according to the measurement site. However, a lower reference wind velocity exhibited a corresponding larger rate of decline of wind velocities. In addition, in terms of wind angle changes, the velocities of winds blown from sloped and valley-shaped areas decreased by up to over 60% after the construction of the embankment. In addition, the rate of decline of wind velocities due to the level difference of surrounding areas according to the embankment's height was found to be largest in the area with the largest level difference from the central part of the embankment. Wind velocity changes by the distance from the embankment exhibited an increase in the decline range of wind velocities up to the distance of 3*H*. Field measurements were conducted to determine wind velocity and temperature changes after the construction of the embankment. The results of the field measurements also confirmed wind velocity changes according to the embankment's height and level difference. In the central part of the embankment, the lowest wind velocity was measured, whereas the degree of wind velocity change was found to be small. The results of the wind tunnel test were in line with the general tendency. The site with small wind velocity changes (below the embankment) recorded lower temperatures than the higher site. Temperature changes in the evening and at dawn when low wind velocities were measured were larger compared to other hours. After the construction of the embankment, temperatures also dropped along with wind velocities.
